# A proteomic approach for studying insect phylogeny: CAPA peptides of ancient insect taxa (Dictyoptera, Blattoptera) as a test case

**DOI:** 10.1186/1471-2148-9-50

**Published:** 2009-03-03

**Authors:** Steffen Roth, Bastian Fromm, Gerd Gäde, Reinhard Predel

**Affiliations:** 1 Institute of Zoology, University of Jena, Erbertstrasse 1, D-07743 Jena, Germany; 2 Zoology Department, University of Cape Town, Rondebosch 7701, South Africa; 3 Institute of Biology, University of Bergen, Bergen N-5020, Norway

## Abstract

**Background:**

Neuropeptide ligands have to fit exactly into their respective receptors and thus the evolution of the coding regions of their genes is constrained and may be strongly conserved. As such, they may be suitable for the reconstruction of phylogenetic relationships within higher taxa. CAPA peptides of major lineages of cockroaches (Blaberidae, Blattellidae, Blattidae, Polyphagidae, Cryptocercidae) and of the termite *Mastotermes darwiniensis *were chosen to test the above hypothesis. The phylogenetic relationships within various groups of the taxon Dictyoptera (praying mantids, termites and cockroaches) are still highly disputed.

**Results:**

Tandem mass spectrometry of neuropeptides from perisympathetic organs was used to obtain sequence data of CAPA peptides from single specimens; the data were analysed by Maximum Parsimony and Bayesian Interference. The resulting cladograms, taking 61 species into account, show a topology which is in general agreement with recent molecular and morphological phylogenetic analyses, including the recent phylogenetic arrangement placing termites within the cockroaches. When sequence data sets from other neuropeptides, viz. adipokinetic hormones and sulfakinins, were included, the general topology of the cladogram did not change but bootstrap values increased considerably.

**Conclusion:**

This study represents the first comprehensive survey of neuropeptides of insects for solely phylogenetic purposes and concludes that sequences of short neuropeptides are suitable to complement molecular biological and morphological data for the reconstruction of phylogenetic relationships.

## Background

Peptides are short proteins, whose power to resolve phylogenetic questions have already been recognized (e.g. [1,2]). Peptide mass fingerprints support species recognition in many cases, particularly in organisms that exhibit few morphological differences such as microorganisms [3]. A specific group of peptides are the neuropeptides, structurally diverse messenger molecules, which influence a wide-range of physiological processes [4]. Due to their role as ligands, which have to fit into the respective receptors, neuropeptides are under considerable evolutionary constraint. Consequently, the regions of neuropeptide genes encoding for mature peptides may be highly conserved and suitable for the reconstruction of deep level phylogenetic relationships within higher taxa. However, only few attempts have been made to use these substances for phylogenetic purposes. Gäde [5] first introduced this approach for neuropeptides belonging to adipokinetic/hypertrehalosaemic hormones. The few sequence variations of these hormones within insects, however, do not contain sufficient information for a detailed analysis of phylogenetic relationships, although grouping of certain taxa is possible [6,7]. Other peptide families with multiple forms such as allatostatins [8] have both conserved and fast-evolving peptide sequences and are certainly more significant in this context but less extensively studied. The conserved sequences may be suitable for the reconstruction of phylogenetic relationships within higher taxa and the fast-evolving sequences may be more suitable for the reconstruction of tip-level phylogenetic relationships within closely related taxa.

Conducting a phylogenetic analysis of the genes encoding neuropeptides is not an easy task. In most cases, only small portions of these genes have been highly conserved, specifically the regions encoding for mature peptides, which interact with their receptors. Thus, primers successfully used for the identification of neuropeptide genes in a certain insect species may fail to recognize the orthologous gene in a related species (Derst, Roth, Predel; unpublished). Recent developments in mass spectrometric techniques [9], however, have paved the way for a rapid identification of mature neuropeptides from single insect specimens [10-13], thereby circumventing the genomic approach.

In the present study, tandem mass spectrometry was used for the first time to perform an extensive phylogenetic study on neuropeptides of insects, focusing on CAPA peptides of Dictyoptera. CAPA peptides were first identified from the American cockroach, *Periplaneta americana *[14-16]. CAPA-genes are known from a number of holometabolous insects (e.g. *Drosophila melanogaster*: [17], *Anopheles gambiae*: [18], *Apis mellifera*: [19], *Tribolium castaneum*: [20]). These genes encode for up to four peptides, which belong to CAPA-periviscerokinins (PVKs) and CAPA-pyrokinins (PKs). Both groups of CAPA peptides bind to different receptor types [21,22]. Besides their expression in a few interneurons, CAPA peptides are always part of the neuroendocrine system of the abdominal ventral nerve cord and are likely released into the haemolymph via abdominal perisympathetic organs (PSOs). Direct mass spectrometric screening of these organs (see [10,23]) allowed the unambiguous identification of the CAPA peptides from single specimens and cleared the way for a large-scale screening of these neuropeptides in the taxon Dictyoptera.

The taxon Dictyoptera includes praying mantids (Mantodea), termites (Isoptera), and cockroaches (Blattoptera) (e.g. [24]), and members are among the oldest pterygote insects known. Both morphological and molecular data support a monophyly of Mantodea and Isoptera (see [25]). The relationships of Mantodea, Isoptera, and Blattoptera, the monophyly of Blattoptera and the relationships among several cockroach lineages are, however, a topic of conflicting conclusions (e.g. [26-38]). In particular, the position of the genus *Cryptocercus *within the Blattoptera and its relationship with Isoptera has been the focus of numerous phylogenetic studies. Grandcolas (analysis of morpho-anatomical data: [39,40]) and Gäde et al. (analysis of adipokinetic hormones: [6]) placed these wood-feeding cockroaches in the Polyphagidae. Molecular data, however, suggest a sister-relationship between termites and *Cryptocercus *[41-44], a historical position [45] that is supported by Deitz et al. [46], and Klass & Meier [47] based on morpho-anatomical data. Inward et al. [44] presented convincing data to suggest that Isoptera nest within Blattoptera. The monophyly of several cockroach taxa and subgroups of these taxa is, however, doubtful. In a recent analysis of five gene loci, Inward et al. [44] found no support for the monophyly of the Blattellidae and subordinated taxa within the Blaberidae. In some of these taxa, further data acquisition of conventional molecular and morphological characters and more species may provide sufficient information to resolve more precisely the phylogenetic relationship of certain taxa within the Blattoptera (see [44,47]). In cases where these attempts result in conflicting hypotheses about the placement and monophyly of different taxa, additional characters (e.g. sequences of neuropeptides) may be required to test the robustness of the different analyses.

To test the phylogenetic information of neuropeptides in general, we used a stepwise approach by analysing the topology and stability of the phylogenetic trees, starting with the CAPA peptide data set followed by repeated analyses with additional neuropeptide sequences, namely adipokinetic hormone (AKH-1) and sulfakinins (SKs).

The cladograms obtained from these peptide sequences confirmed that certain neuropeptide sequences of insects are able to complement molecular, biological and morphological data for the reconstruction of phylogenetic relationships.

## Results

### Data acquisition and alignment

**Figure 1:**
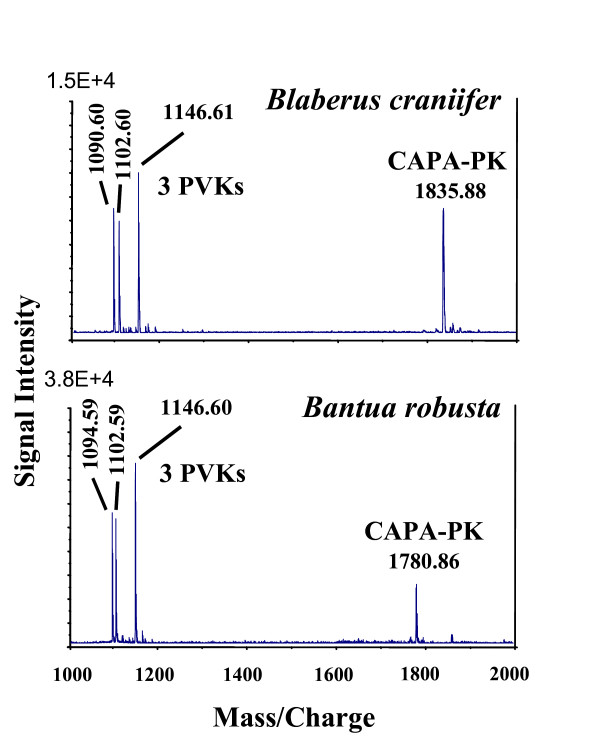
**Comparison of MALDI-TOF mass spectra (mass range 1000–2000 Da) of single abdominal PSO preparations of *Blaberus craniifer *and *Bantua robusta *(= peptide hormone fingerprint)**. Only few abundant substances are detectable. Underlying sequences were used for phylogenetic analyses.

**Figure 2:**
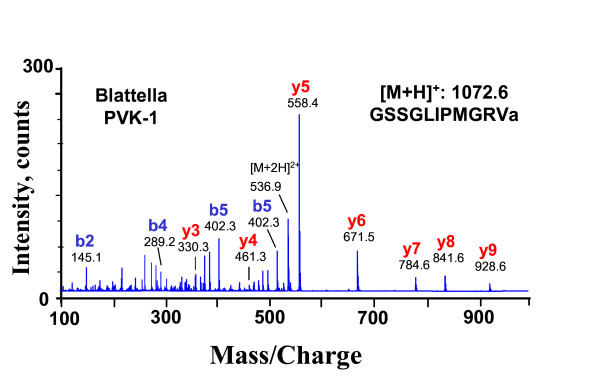
**CID spectrum (ESI-QTOF MS) of *Blattella germanica *PVK-1 at [M+2H]^2+ ^536.9 ([M+H]^+^:1072.6)**. The y- and b-type fragment ions are labelled. Fragments were analyzed manually and the resulting sequence is given in the inset.

Direct mass spectrometric analysis of abdominal PSO preparations of single specimens (examples given in Figures 1, 2) revealed complete sequences of CAPA peptides from 61 cockroach/termite species. The species list covers major taxa of cockroaches (Blattidae, Polyphagidae, Cryptocercidae, Blaberidae, Blattellidae) and the termite *Mastotermes darwiniensis*. From most species, three CAPA-periviscerokinins (PVKs), and a single CAPA-pyrokinin (PK) were sequenced. *Cryptocercus *and the blattellid cockroaches *Symploce pallens *and *Loboptera decipiens *express only two different PVKs. A fourth PVK (designated PVK-4) was found in the Madagascan Blaberidae and the Table Mountain cockroach *Aptera fusca *(for sequences see [48]. These PVK-4 peptides, whose sequences suggest an internal gene duplication of PVK-1 (*Elliptorhina*, *Gromphadorhina*, *Princisia*) or PVK-2 (*Aptera*), did not influence the topology of phylogenetic trees and were not included in the final alignments. The average size of the PVKs was 11 amino acids (aa) and that of the PK was 17 aa. Sequences of the CAPA peptides were combined for each species and aligned (Table 1). With the inclusion of gaps and sequences of the outgroup species (*Locusta migratoria *and *Drosophila melanogaster*), the alignment resulted in 58 characters. Thirteen characters were constant, 12 variable characters were parsimony-uninformative, and 33 variable characters were parsimony-informative. The sequence of PVK-2 was found to be highly conserved and did not contain phylogenetically informative substitutions.

**Table 1:** Sequences of CAPA peptides aligned with Clustal X

**Species**	**CAPA-PVK1**	**CAPA-PVK2**	**CAPA-PVK3**	**CAPA-PK**
*Ergaula capucina*	GSS-GLISFPRTa	GS-SGLISMPRVa	--QLG-L-PFPRVa	SASG-SGESSGMWFGPRLa
*Polyphaga aegyptiaca*	GTS-GLISFPRTa	GS-SGLISMPRVa	--QVG-LIPFPRVa	SASGGAGESSGMWFGPRLa
*Blatta orientalis*	GAS-GLIPVMRNa	GS-SGLISMPRVa	GSSSG-LISMPRVa	GGGG-SGETSGMWFGPRLa
*Neostylopyga rhombifolia*	GAS-GLIPVMRNa	GS-SGLISMPRVa	GSSSG-LISMPRVa	GGGG-SGETSGMWFGPRLa
*Periplaneta americana*	GAS-GLIPVMRNa	GS-SGLISMPRVa	GSSSG-LISMPRVa	GGGG-SGETSGMWFGPRLa
*Periplaneta australasiae*	GAS-GLIPVMRNa	GS-SGLISMPRVa	GSSSG-LISMPRVa	GGGG-SGETSGMWFGPRLa
*Periplaneta brunnea*	GAS-GLIPVMRNa	GS-SGLISMPRVa	GSSSG-LISMPRVa	GGGG-SGETSGMWFGPRLa
*Periplaneta fuliginosa*	GAS-GLIPVMRNa	GS-SGLISMPRVa	GSSSG-LISMPRVa	GGGG-SGETSGMWFGPRLa
*Pseudoderopeltis bimaculata*	GAS-GLIPVMRNa	GS-SGLISMPRVa	GSSSG-LISMPRVa	GGGG-SGETSGMWFGPRLa
*Shelfordella lateralis*	GAS-GLIPVMRNa	GS-SGLISMPRVa	GSSSG-LISMPRVa	GGGG-SGETSGMWFGPRLa
*Deropeltis erythrocephala*	GAS-GLIPVMRNa	GS-SGLISMPRVa	GGSSG-LISMPRVa	GGGG-SGETSGMWFGPRLa
*Deropeltis atra*	GAS-GLIPVMRNa	GS-SGLISMPRVa	GGSSG-LISMPRVa	GGGG-SGETSGMWFGPRLa
*Deropeltis integerrima*	GAS-GLIPVMRNa	GS-SGLISMPRVa	GGSSG-LISMPRVa	GGGG-SGETSGMWFGPRLa
*Pseudoderopeltis flavescens*	GAS-GLIPVMRNa	GS-SGLISMPRVa	GGSSG-LISMPRVa	GGGG-SGETSGMWFGPRLa
*Pseudoderopeltis foveolata*	GAS-GLIPVMRNa	GS-SGLISMPRVa	GGSSG-LISMPRVa	GGGG-SGETSGMWFGPRLa
*Eurycotis floridana*	GAS-GLIPVMRNa	GS-SGLISVPRVa	GGSSG-LISVPRVa	GGGG-SGETSGMWFGPRLa
*Cryptocercus darwini*	?????????????	GS-SGLISMPRVa	G-SSG-LIAMPRVa	GGGG-SGETSGMWFGPRLa
*Cryptocercus kyebangensis*	?????????????	GS-SGLISMPRVa	G-SSG-LIAMPRVa	EGSG-SGETSGMWFGPRLa
*Mastotermes darwiniensis*	ASS-GLISMPRVa	GS-SGLIPMPRVa	S-SSG-LIPMPRVa	GGSG-SGETSGMWFGPRLa
*Therea petiveriana*	GSS-GLISFPRNa	GS-SGLISMPRVa	G-SSG-LISMTRVa	SASG-SGESSGMWFGPRLa
*Gyna lurida*	GST-GLIPFGRTa	GS-SGLISMPRVa	G-SSG-MIPFPRVa	AGDT-SSEAKGMWFGPRLa
*Gyna caffrorum*	GST-GLIPFGRTa	GS-SGLISMPRVa	G-SSG-MIPFPRVa	AGDT-SSEAKGMWFGPRLa
*Aptera fusca*	GSS-GLIPFGRTa	GS-SGLISMPRVa	G-SSG-IIPFPRVa	SGDT-SSQAKGMWFGPRLa
*Blaberus craniifer*	GSS-GLIPFGRTa	GS-SGLISMPRVa	G-SSG-MIPFPRVa	AGES-SNEAKGMWFGPRLa
*Blaberus giganteus*	GSS-GLIPFGRTa	GS-SGLISMPRVa	G-SSG-MIPFPRVa	AGES-SNEAKGMWFGPRLa
*Eublaberus distanti*	GSS-GLIPFGRTa	GS-SGLISMPRVa	G-SSG-MIPFPRVa	AGES-SNEAKGMWFGPRLa
*Eublaberus posticus*	GSS-GLIPFGRTa	GS-SGLISMPRVa	G-SSG-MIPFPRVa	AGES-SNEAKGMWFGPRLa
*Eublaberus spec*.	GSS-GLIPFGRTa	GS-SGLISMPRVa	G-SSG-MIPFPRVa	AGES-SNEAKGMWFGPRLa
*Blaptica dubia*	GSS-GLIPFGRTa	GS-SGLISMPRVa	G-SSG-MIPFPRVa	GGES-SNEAKGMWFGPRLa
*Lucihormetica grossei*	GST-GLIPFGRTa	GS-SGLISMPRVa	G-SSG-MIPFPRVa	GGES-SNEAKGMWFGPRLa
*Lucihormetica subcincta*	GST-GLIPFGRTa	GS-SGLISMPRVa	G-SSG-MIPFPRVa	GGES-SNEAKGMWFGPRLa
*Lucihormetica verrucosa*	GST-GLIPFGRTa	GS-SGLISMPRVa	G-SSG-MIPFPRVa	GGES-SNEAKGMWFGPRLa
*Archimandrita tesselata*	GSS-GLIPFGRTa	GS-SGLISMPRVa	G-SSG-MIPFPRVa	EGAN-SNEAKGMWFGPRLa
*Panchlora spec*.	GSS-GLIPMGRTa	GS-SGLISMPRVa	G-SSG-MIPFPRVa	GGET-GNDAKAMWFGPRLa
*Panchlora viridis*	GSS-GLIPMGRTa	GS-SGLISMPRVa	G-SSGGMIPFPRVa	GGET-GSDAKAMWFGPRLa
*Cyrtotria poduriformis*	GST-GLIPFGRTa	GS-SGLISMPRVa	G-SSG-MIPFPRVa	SGET-SGEGNGMWFGPRLa
*Hostilia carinata*	GST-GLIPFGRTa	GS-SGLISMPRVa	G-SSG-MIPFPRVa	SGET-SGEGNGMWFGPRLa
*Perisphaeria aff. bicolor*	GST-GLIPFGRTa	GS-SGLISMPRVa	G-SSG-MIPFPRVa	SGET-SGEGNGMWFGPRLa
*Pilema dubia*	GST-GLIPFGRTa	GS-SGLISMPRVa	G-SSG-MIPFPRVa	SGET-SGEGNGMWFGPRLa
*Perisphaeria substylifera*	GST-GLIPFGRTa	GS-SGLISMPRVa	G-SSG-MIPFPRVa	SGET-SGEGNGMWFGPRLa
*Perisphaeria scabrella*	GST-GLIPFGRTa	GS-SGLISMPRVa	G-SSG-MIPFPRVa	SGET-SGEGNGMWFGPRLa
*Blepharodera discoidalis*	GST-GLIPFGRTa	GS-SGLISMPRVa	G-SSG-MIPFPRVa	SGET-SGEGNGMWFGPRLa
*Perisphaeria virescens*	GST-GLIPFGRPa	GS-SGLISMPRVa	G-SSG-MIPFPRVa	SGET-SGEGNGMWFGPRLa
*Bantua robusta*	GST-GLISFGRTa	GS-SGLISMPRVa	G-SSG-MIPFPRVa	SGET-SGEGNGMWFGPRLa
*Diploptera punctata*	GSS-GLIPFGRTa	GS-SGLISMPRVa	G-SSG-MIPFPRVa	SGET-SGEGNGMWFGPRLa
*Perisphaeria ruficornis*	GSS-GLIPFGRTa	GS-SGLISMPRVa	G-SSG-LIPFPRVa	SGET-SGEGNGMWFGPRLa
*Elliptorhina spec*.	GSS-GLIPFGRTa	GS-SGLISMPRVa	G-SSG-MIPFPRVa	FGET-SGETKGMWFGPRLa
*Gromphadorhina portentosa*	GSS-GLIPFGRTa	GS-SGLISMPRVa	G-SSG-MIPFPRVa	FGET-SGETKGMWFGPRLa
*Gromphadorhina grandidieri*	GSS-GLIPFGRTa	GS-SGLISMPRVa	G-SSG-MIPFPRVa	FGET-SGETKGMWFGPRLa
*Princisia vanwaerenbeki*	GSS-GLIPFGRTa	GS-SGLISMPRVa	G-SSG-MIPFPRVa	FGET-SGETKGMWFGPRLa
*Rhyparobia maderae*	GSS-GLIPFGRTa	GS-SGLISMPRVa	G-SSG-MIPFPRVa	FGET-SGETKGMWFGPRLa
*Laxta *spec.	GST-GLIPFGRTa	GS-SGLISMPRVa	G-SSG-MIPFPRVa	GGET-SGETKGMWFGPRLa
*Pycnoscelus surinamensis*	GSP-GLIPFGRSa	GS-SGLISMPRVa	G-SSG-MIPFPRVa	GGET-SGEGKGMWFGPRLa
*Derocalymma cruralis*	GSSGGLITFGRTa	GSLTGLISMPRTa	G-SSG-MISFPRTa	DGDM-SGEGKGMWFGPRLa
*Derocalymma versicolor*	GSSGGLITFGRTa	GSLTGLISMPRTa	G-SSG-MISFPRTa	TGDM-SGEGKGMWFGPRLa
*Panesthia spec*.	GSS-GLISFPRVa	GS-SGLISMPRVa	G-SSG-MIPFPRVa	GGET-SGEGKGMWFGPRLa
*Blattella germanica*	GSS-GLIPMGRVa	GS-SGLISMPRVa	G-SSG-MIPFPRVa	ESGG-SGEANGMWFGPRLa
*Loboptera decipiens*	?????????????	GS-SGLISMPRVa	G-SSG-MIPFPRVa	GSGG-SGEANGMWFGPRLa
*Supella dimidiata*	GSS-GLIAMPRVa	GS-SGLISMPRVa	G-SSG-MIPFPRVa	GGGS-SGETNGMWFGPRLa
*Supella longipalpa*	GSS-GLIAMPRVa	GS-SGLISMPRVa	G-SSG-MIPFPRVa	GGGS-SGETNGMWFGPRLa
*Symploce pallens*	?????????????	GS-SGLISMPRVa	G-SSG-MIPFPRVa	EGGS-SGEASGMWFGPRLa
*Drosophila melanogaster*	-------------	AS--GLVAFPRVa	GANMG-LYAFPRVa	TGPS---ASSGLWFGPRLa
*Locusta migratoria*	-AA-GLFQFPRVa	----GLLAFPRVa	TSS---LFPHPRLa	DGGE---PAAPLWFGPRVa

### Sequence variation of CAPA peptides within and among populations

We did not observe a single sequence variation of CAPA peptides from males, females, and larvae within any of the cockroach populations investigated. The PSOs of the American cockroach, *P. americana*, served as control in most mass spectrometric analyses (n > 400), and there was a lack of variability of neuropeptides at the individual level. We compared the CAPA peptides for a number of species (*Diploptera punctata, Loboptera decipiens, Blaberus craniifer*) that had been raised in a culture for multiple generations with specimens collected in the field. In addition, three South African populations of *Bantua robusta *that were collected in the rainforest (Tsitsikamma), fynbos (Cape Town), and Karroo vegetation (Kamieskroon) were investigated but no sequence variations were found (data not shown).

### Analysis of phylogenetic relationships by means of CAPA sequences

**Figure 3:**
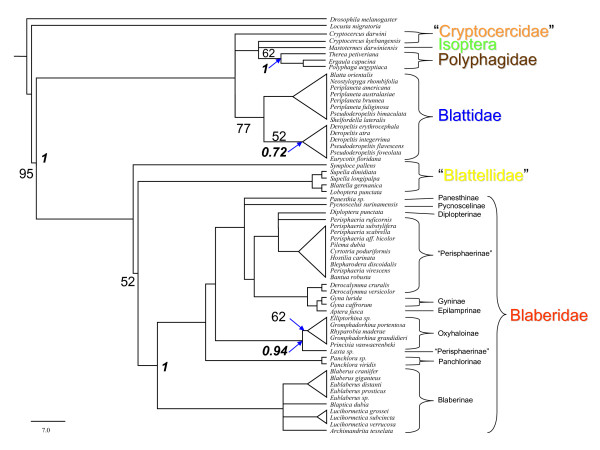
**Phylogenetic relationships of cockroaches based on CAPA peptide sequences represented by a maximum parsimony (MP) 50% majority rules consensus tree**. Numbers on the branches indicate bootstrap values (≥ 50) for MP. Italic numbers on the nodes indicate posterior probability values (≥ 0.5) (proportion of the 18205 sampled trees that contain the node). Tree length = 142, Consistency index (CI) = 0.768, Homoplasy Index (HI) = 0.232, Retention index (RI) = 0.907, Rescaled consistency index (RC) = 0.696.

Due to the high level of conservation in the sequences of PVK-2 as well as in the C-termini of the other CAPA peptides, only 33 amino acid positions contained phylogenetically informative characters. It was intriguing to see that the Maximum Parsimony (MP) analysis (Figure 3) obtained from these data was generally in agreement with recent molecular [44] and morphological [47] analyses, although the bootstrap values were relatively low. Significant support (bootstrapping, posterior probabilities of Bayesian analysis) was found for the monophyly of Blaberoidea (Blattellidae + Blaberidae) and Blattidae. The cladograms also support sister-group relationships between Blaberoidea and Blattoidea, Blattellidae and Blaberidae, and Blattidae and Polyphagidae + Cryptocercidae + *Mastotermes*. Within the latter clade, the three polyphagid species (*Polyphaga aegyptiaca, Ergaula capucina, Therea petiveriana*) appear as a monophyletic group separated from an unsolved sister-group containing *Cryptocercus kyebangensis *and *Mastotermes darwiniensis*. A Bayesian consensus tree (see additional file 1): Phylogenetic relationships based on neuropeptide sequences represented by a Bayesian majority rules consensus tree) yielded almost identical topologies with those that were obtained from Maximum Parsimony.

Although the relationships within the Blaberidae (members of 9 of 11 subfamilies were included in this study) were poorly resolved, the different clades comprised, with few exceptions, only members of specific subfamilies. This was found for Blaberinae (*Blaberus, Eublaberus, Lucihormetica, Archimandrita *and *Blaptica*), Oxyhaloinae (Madagascan genera *Princisia, Elliptorhina, Gromphadorhina *as well as *Rhyparobia*), Panchlorinae (*Panchlora *species), and Perisphaeriinae (Southern African *Cyrtotria*, *Perisphaeria*, *Bantua*, *Hostilia*, and *Pilema*). In contrast, monophyly was not supported for some genera which are currently grouped in the Perisphaeriinae (see [49]). The Australian genus *Laxta *and African genus *Derocalymma*, both containing extremely flattened cockroaches which are adapted for living under bark, did not show close relationships with each other or with the remaining Perisphaeriinae. Instead, *Derocalymma *was found in a clade also containing *Gyna *and the Table Mountain cockroach, *Aptera fusca*. *Blepharodera discoidalis*, which was removed from the Perisphaeriinae by Grandcolas [49], contained CAPA peptides typical of Perisphaeriinae.

**Figure 4:**
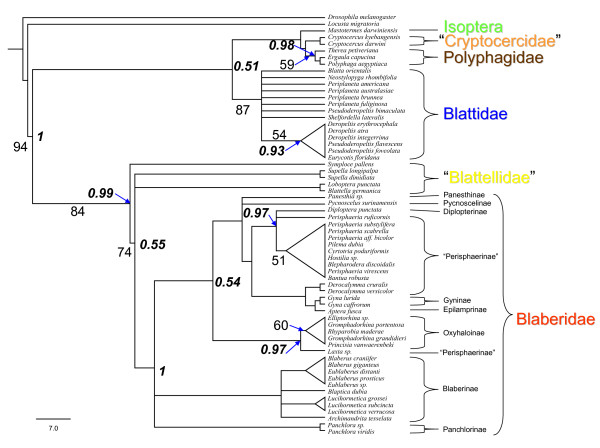
**Phylogenetic relationships of cockroaches based on CAPA peptides, AKH-1 and sulfakinin sequences represented by a maximum parsimony (MP) 50% majority rules consensus tree**. Numbers on the branches indicate bootstrap values (≥ 50) for MP. Italic numbers on the nodes indicate posterior probability values (≥ 0.5) (proportion of the 20206 sampled trees that contain the node). Tree length = 181, Consistency index (CI) = 0.796, Homoplasy Index (HI) = 0.204, Retention index (RI) = 0.917, Rescaled consistency index (RC) = 0.729.

To test if the topology of the phylogenetic trees remains stable, the phylogenetic analysis was repeated with additional neuropeptide sequences, namely adipokinetic hormone (AKH-1) and sulfakinins (SKs) (see [50]). These peptides are stored in the corpora cardiaca, and mass fingerprints from these organs were sufficient for the correct assignment of the group-specific sequences in all cases. The resulting cladograms confirmed the topology of the former analysis, and increased the bootstrap values (Figure 4 and see additional file 2): Phylogenetic relationships based on peptide sequences represented by a Bayesian majority rules consensus tree).

## Discussion

The current investigation represents the first comprehensive survey of neuropeptides of insects for entirely phylogenetic purposes. Although the introduction of novel characters is consistently requested to corroborate existing hypotheses on phylogenetic relationships in insects (see [51]), such new character sets and methods have to compete with well-established methods. In order for our methodological approach to be acceptable by systematists using established methods, we developed techniques that allowed us to sample sufficient taxa and perform the analysis quickly.

In recent years, MALDI-TOF mass spectrometric analysis has been routinely used for studying the peptidome of the neuroendocrine system of insects [11-13,51-54]. The power of modern mass spectrometry means that only a few specimens of insects as small as the red flour beetle *Tribolium castaneum *are necessary to confirm the expression of more than 60 neuropeptides when genome information is available [20]. In the present study, however, genome information was not available, and the homologous peptides of the different species had to be *de-novo *sequenced. This approach posed a bioanalytical challenge and required a decision about the neuropeptide species to be included before extensive taxon sampling. The decision to select CAPA peptides first (see [48]) was made because these peptides fulfil certain criteria for a successful reconstruction of phylogenetic relationships. First, these peptides occur at high concentrations in neurohaemal tissues (abdominal PSOs), which are fairly easy to dissect, do not contain other neuropeptides at high concentrations and, thus, allow sequence elucidation from PSOs from a single specimen. Moreover, the detection of specific neuropeptide gene products, such as CAPA peptides, from defined neurohaemal organs usually excludes the alignment of peptides with sequence similarities that result from convergent evolution (homoplasy). Second, multiple members of related peptides encoded by single genes exist in insects. If the number of these often closely related peptide paralogues differs between related species, alignments may become difficult. Hence, it is more convenient to use a peptide family that contains the same number of peptide forms in the taxa of interest. In such a case, the storage organ as well as the conserved sequences of the peptide hormones can be used to assign the homologous peptides. Several peptide families were initially included in preliminary experiments; the CAPA peptides met the aforementioned criteria best and were thus used for this phylogenetic study. Since the sequence information from these peptides spans a length of 50 amino acids only, the resulting phylogenetic tree shows low posterior probabilities and low bootstrap levels.

In a subsequent and very rapid experimental approach, we used mass fingerprint data to include further neuropeptide sequences from relatively conserved peptides (AKH-1 and sulfakinins) in the phylogenetic analyses. The resulting topology of the cladograms did not change but the bootstrap values increased considerably. Since the additional neuropeptides did not differ very much between closely related taxa or did not differ at all, bootstrap levels of higher taxa were higher than those within lower taxa. This supports the hypothesis that, as a result of the decelerated co-evolution of neuropeptides and their receptors, neuropeptide sequences may be particularly suitable for the reconstruction of phylogenetic relationships within higher taxa.

The cladograms in Figures 3 and 4 show a topology that is in general agreement with recent molecular [44] and morphological phylogenetic analyses [47], including the recent phylogenetic arrangement placing termites within the cockroaches. Questions arising from the current data are: how can we solve existing polytomies, how can we enhance bootstrap supports for existing clades, and how can we possibly extend the analysis to higher or lower taxa? Sampling more taxa and only analysing CAPA peptides, AKHs and sulfakinins is unlikely to provide sufficient data to solve all of these questions. A combination of well chosen taxa sampling (including the outgroup taxa) and other neuropeptides will be needed to solve the relationship among the major lineages of Dictyoptera.

At a lower taxonomic level, however, a higher number of analyzed species in well-defined groups (e.g. Perisphaeriinae) may provide sufficient information to re-assess the generic composition of that group. Our data regarding the Perisphaeriinae differ, in part, from the suggestions made by Grandcolas [49], who analyzed head morphology and genitalia. The data do not support the removal of *Blepharodera *from this subfamily (see also [55]), and do not verify a close relatedness of *Derocalymma *and *Laxta *with the other genera of Perisphaeriinae. Indeed, we found six genera of Perisphaeriinae with completely identical neuropeptide sequences (*Perisphaeria, Blepharodera, Pilema, Hostilia, Bantua*, *Cyrtotria*) and these are exactly the genera which were placed in a single tribe (Perisphaeriini) by Roth [56].

We did not test how the choice of outgroup and ingroup taxa affects tree topology but further taxon sampling seems to be essential in termites and blattellid cockroaches. For the latter taxon, we have already obtained partial sequences from further species (unpublished data), which support the para- or polyphyletic origin of this group. In most cockroach groups (e.g. Blaberidae), however, even a more representative and comprehensive incorporation of further taxa is unlikely to provide novel insights into phylogenetic relationships. In these cases, further peptide families have to be included for phylogenetic analyses. In the present initial attempt, seven homologous neuropeptides of 61 species of Blattoptera were tested.

**Figure 5:**
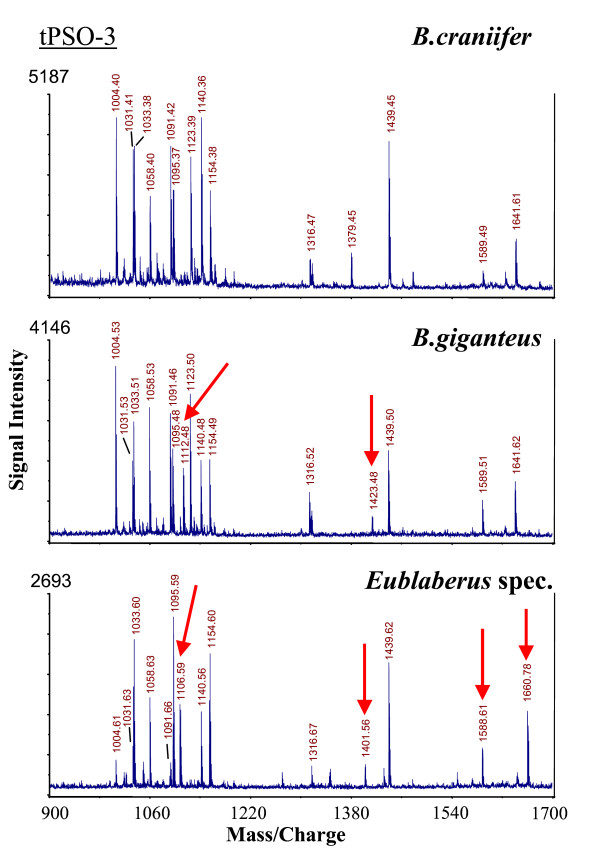
**MALDI-TOF mass spectra (neuropeptide mass fingerprints) from single thoracic PSO preparations of three *Blaberus/Eublaberus *species, representing FMRFamide related peptides which accumulate in the neurohaemal organs of insects (see Predel et al. 2004)**. The selected species were not distinguishable by screening the CAPA peptides from abdominal PSOs. All ion signals different from those of *B. craniifer *are marked. Such fingerprint data exist from all neurohaemal organs of all cockroach species investigated.

From a single cockroach, *P. americana*, roughly 80 neuropeptides have been elucidated by biochemical methods in recent years. Today, most of these peptides can be identified by mass spectrometric techniques as described in this manuscript, which makes these peptides generally suitable for phylogenetic studies. Fast evolving neuropeptides such as FMRFamides [57] can provide phylogenetic information at the generic level (see Figure 5) [58] but are not suitable for studying the deep level relationships of higher taxa within an insect order because the homology of such peptide copies among far related taxa can be difficult to assess. Other peptide families with multiple members, such as tachykinin-related peptides, pyrokinins, and allatostatins [59] are likely to be most suitable for the incorporation in phylogenetic analyses. These peptide families are represented by more than 30 paralogues in *P. americana*. Previous experiments have already shown that members of the Blattidae, which cannot be further separated from each other by the analysis of CAPA peptides, AKHs, and sulfakinins, are clearly distinguishable if species-specific pyrokinin sequences are identified [23]. These findings confirm that even short neuropeptide sequences of insects are suitable to complement molecular biological and morphological data for the reconstruction of phylogenetic relationships.

## Conclusion

The phylogenetic relationships within the major lineages of cockroaches (Blaberidae, Blattellidae, Blattidae, Polyphagidae, Cryptocercidae) and their relationship to termites (Isoptera) were reconstructed by using the first comprehensive survey of neuropeptides of insects for solely phylogenetic purposes. The cladograms resulting from the analysis of peptide sequences of 61 Blattoptera species show a topology which is in general agreement with recent molecular and morphological phylogenetic analyses and also confirm the grouping of Isoptera within Blattoptera. Regarding other hypotheses about cockroach phylogeny, our data support the monophyly of Blaberoidea (Blattellidae + Blaberidae) and Blattidae. The cladograms also support sister-group relationships between Blaberoidea and a monophylum of the remaining cockroaches (including Isoptera), paraphyletic Blattellidae and Blaberidae, and Blattidae and Polyphagidae + Cryptocercidae + *Mastotermes*. This study verified that sequences of several neuropeptide families can complement molecular biological and morphological data for the reconstruction of phylogenetic relationships.

## Methods

### (a) Insects

In total, 61 species of Dictyoptera, representing the five cockroach taxa Polyphagidae, Cryptocercidae, Blattidae, Blattellidae, Blaberidae, and the termite *Mastotermes darwiniensis *were analyzed. *Locusta migratoria *(Orthoptera) and *Drosophila melanogaster *(Diptera) were used as outgroup species; the CAPA peptides of these species were identified by Predel & Gäde [60], Clynen et al. [61] and Kean et al. [17]. The names and places of collection (or sources of cockroach/termite cultures) of all species examined in this study, as well as the SWISSPROT accession numbers for peptide sequences are given in additional file 3. For most of the species, a mass fingerprint which represented about 40 peptide hormones was obtained from the major hormone release sites (corpora cardiaca, thoracic and abdominal perisympathetic organs). The respective fingerprints are typical of very closely related species (see Figure 5) and may be species-specific (see [62,63]). Remains of the insects as well as the fingerprint data can be obtained from the corresponding author.

### (b) Mass spectrometry

The dissection of the neurohaemal organs (abdominal perisympathetic organs, corpora cardiaca) as well as the sample preparation for MALDI-TOF MS (matrix-assisted laser desorption ionization time-of-flight mass spectrometry) and ESI-QTOF MS (electrospray ionization time-of-flight mass spectrometry) were performed as previously described [11,57]. *MALDI-TOF MS*: Mass spectra were obtained using an ABI 4700 proteomics analyzer (Applied Biosystems, Framingham, MA). To determine the sequences of the peptides, tandem MS experiments with a CID (collision induced dissociation) acceleration of 1 kV were performed. An unambiguous assignment of internal Leu/Ile was achieved by means of CID under high gas pressure that revealed unique patterns for the side chains of Leu and Ile (see [64]). Samples with CAPA peptides that contained Lys/Gln ambiguities were analysed again after dissolving the respective abdominal PSO preparations in acetic anhydride (2:1 methanol/acetic anhydride) which results in rapid acetylation of the ε-amino group of Lys. *ESI-QTOF MS*: In a few cases, data obtained from MALDI-TOF MS did not contain sufficient information to reveal the complete sequences of CAPA peptides. To fill the respective sequence gaps, nanoelectrospray mass spectra were acquired in the positive-ion mode using the API Qstar Pulsar (Applied Biosystems, Applera Deutschland GmbH, Darmstadt, Germany) fitted with a Protana (Odense, Denmark) nanoelectrospray source. Samples were purified using a homemade spin column and analyzed as described in Predel *et al*. [57].

### (c) Sequence alignments and phylogenetic analysis

Homologous peptides were aligned using the Clustal × program package separately (parameter setting: gap penalty = 1; Protein Weight Matrix = BLOSUM), in contrast to aligning the whole data set simultaneously. There was no variability in the alignment results. Assignment of homologous gene products was facilitated due to their storage in specific neurohaemal organs and very similar C-terminal sequences. Phylogenetic analyses of peptides were performed under maximum-parsimony (MP) and Bayesian inference (BI) using PAUP4.0b10 [65] and MrBayes 3.1.2 [66], respectively. In the MP analysis, the heuristic search option with the tree-bisection-reconnection (TBR) branch swapping and 100 stepwise random additions of taxa was used. Gaps corresponding to missing data of few peptides were treated as missing characters, all other gaps as 21^st ^amino acid. Levels of branch support were assessed using bootstrap resampling [67] with 1000 replicates to evaluate the reliability of the inferred topology. In the MP analysis, we tested the different data sets, i.e. CAPA peptides, adipokinetic hormone and sulfakinins, both separately and simultaneously following the total evidence approach. Because the topology of trees was similar (results not shown), we only present the results for our main data set (CAPA peptides) and overall data set. We tested the consistency by calculating the consistency index (CI), retention index (RI), and homoplasy index (HI) (see Figure 3 and 4).

For BI, we analysed the CAPA peptides and complete data set separately by using the fixed rates model test as default in MrBayes. Model free analysis of the peptide data set, however, did not change the topology of the trees (results not shown). A Markov Chain Monte Carlo (MCMC) sampling was run for 1 × 10^6 ^generations and trees were saved every 100 generations (with the first 1000 trees being discarded as "burn-in"). Gaps and missing characters were treated as missing data. Posterior probabilities with values greater than 49% are presented.

## Authors' contributions

The strategy of the paper was mainly developed and coordinated by RP and to some degree by SR. RP and SR have written the manuscript. SR, BF and RP carried out insect dissection, sample preparation, and mass spectrometry; RP was responsible for species identification. BF and SR generated the phylogenetic analysis. Parts of the present study are incorporated within BF's diploma thesis. GG participated in the design and coordination of the study and helped to draft and improve the manuscript. All authors read and approved the final manuscript.

## Supplementary Material

Additional file 1 **Bayesian analysis of CAPA peptides sequences.** Phylogenetic relationships of cockroaches based on CAPA peptides sequences represented by a Bayesian majority rules consensus tree. Numbers on the nodes indicate posterior probability values (≥ 0.49) (proportion of the 18205 sampled trees that contain the node). Click here for file

Additional file 2 **Bayesian analysis of CAPA peptides, AKH-1 and sulfakinin sequences. **Phylogenetic relationships of cockroaches based on CAPA peptides, AKH-1, and sulfakinin sequences represented by a Bayesian majority rules consensus tree. Numbers on the nodes indicate posterior probability values (≥ 0.49) (proportion of the 20206 sampled trees that contain the node). Click here for file

Additional file 3 **Additional information about studied species and accession numbers of peptides.** Information about the species used in this study, including collecting sites/source, accession numbers of CAPA-peptides, AKH-1, sulfakinin-1 to UniProt. The sequence of sulfakinin-2 (Uni-Prot P67802) was identical in all species, except *Loboptera decipiens*, *Symploce pallens *and *Blattella germanica *(sequences not elucidated). Bold accession numbers correspond to sequences identified in this study. For *Drosophila melanogaster *peptides, the Gene-bank accession numbers are given. Click here for file
