# Supplementary figures and images for: A proteomic approach for studying insect phylogeny: CAPA peptides of ancient insect taxa (Dictyoptera, Blattoptera) as a test case
Source: BMC Evol Biol. 2009 Mar 3;9:50. doi: 10.1186/1471-2148-9-50 (PMC2667406; doi:10.1186/1471-2148-9-50)

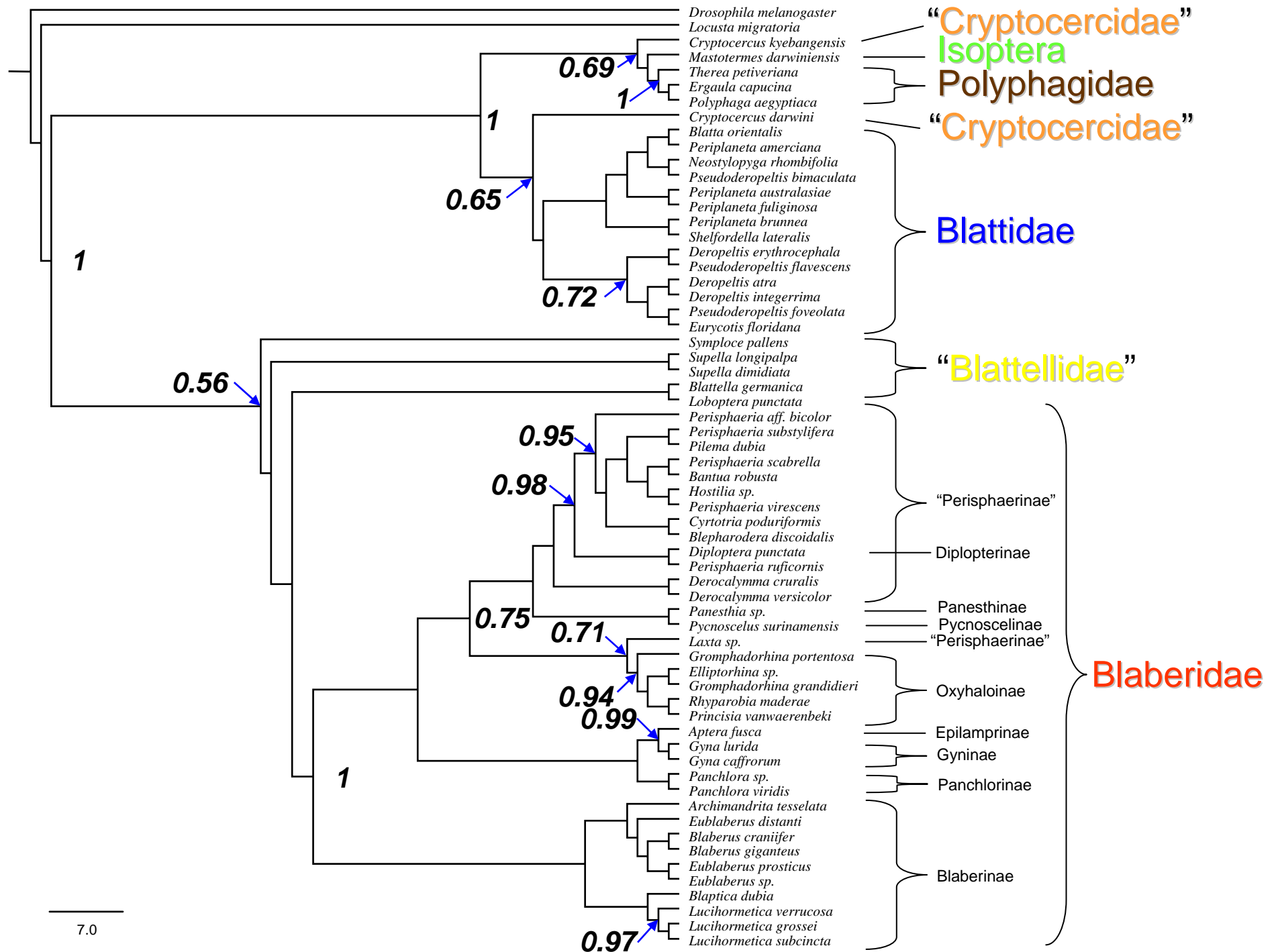

Supplement: Additional file 1 — Bayesian analysis of CAPA peptides sequences. Phylogenetic relationships of cockroaches based on CAPA peptides sequences represented by a Bayesian majority rules consensus tree. Numbers on the nodes indicate posterior probability values (≥ 0.49) (proportion of the 18205 sampled trees that contain the node). [file 1471-2148-9-50-S1.pdf]

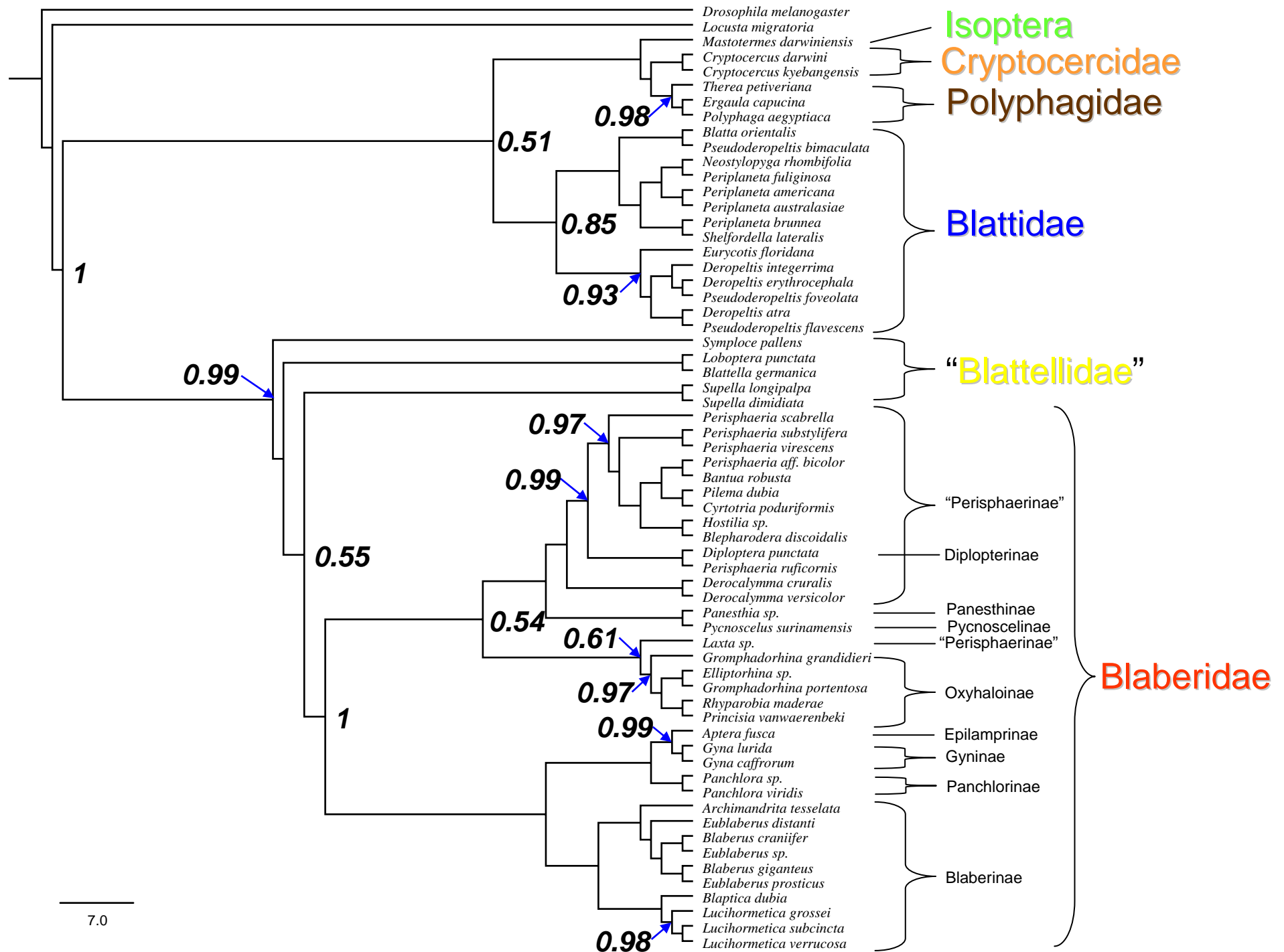

Supplement: Additional file 2 — Bayesian analysis of CAPA peptides, AKH-1 and sulfakinin sequences. Phylogenetic relationships of cockroaches based on CAPA peptides, AKH-1, and sulfakinin sequences represented by a Bayesian majority rules consensus tree. Numbers on the nodes indicate posterior probability values (≥ 0.49) (proportion of the 20206 sampled trees that contain the node). [file 1471-2148-9-50-S2.pdf]
