# Supplementary material for: A proteomic approach for studying insect phylogeny: CAPA peptides of ancient insect taxa (Dictyoptera, Blattoptera) as a test case
Source: BMC Evol Biol. 2009 Mar 3;9:50. doi: 10.1186/1471-2148-9-50 (PMC2667406; doi:10.1186/1471-2148-9-50)
Supplement: Additional file 3 — Additional information about studied species and accession numbers of peptides. Information about the species used in this study, including collecting sites/source, accession numbers of CAPA-peptides, AKH-1, sulfakinin-1 to UniProt. The sequence of sulfakinin-2 (Uni-Prot P67802) was identical in all species, except Loboptera decipiens, Symploce pallens and Blattella germanica (sequences not elucidated). Bold accession numbers correspond to sequences identified in this study. For Drosophila melanogaster peptides, the Gene-bank accession numbers are given. [file 1471-2148-9-50-S3.doc]

| **Species** | **locality/source** | **PVK-1** | **PVK-2** | **PVK-3** | **Capa-PK** | **AKH-1** | **SK-1** |
| --- | --- | --- | --- | --- | --- | --- | --- |
| *Aptera fusca* | Cape Town, Tsitsikamma, ZA | **P85531** | **P85532** | **P84661** | **P84666** | **P85533** | **P85534** |
| *Archimandrita tesselata* | H. Bohn, GER | **P85535** | **P85536** | **P85537** | **P84595** | **P85538** | **P85539** |
| *Bantua robusta* | Kamieskroon, Cape Town, ZA | **P84655** | **P85540** | **P85541** | **P85542** | **P85543** | **P85544** |
| *Blaberus craniifer* | Institute of Zoology Jena, GER | **P83923** | **P83928** | **P83933** | **P85545** | P85546 | **P85547** |
| *Blaberus giganteus* | R. Dusi, GER | **P84591** | **P84592** | **P84593** | **P85556** | P85557 | **submitted** |
| *Blaptica dubia* | Institute of Zoology Jena, GER | **P83924** | **P83929** | **P83934** | **P85548** | **P85549** | **P85550** |
| *Blatta orientalis* | Institute of Zoology Jena, GER | P85558 | P84426 | P84438 | P85559 | P84261 | **P85560** |
| *Blattella germanica* | Institute of Zoology Jena, GER | **P85551** | **P85552** | **P85553** | **P85554** | P84220 | P85555 |
| *Blepharodera discoidalis* | Lutzville, ZA | **P85561** | **P85562** | **P85563** | **P85564** | **P85565** | **P85566** |
| *Cryptocercus darwini* | S. Kambhampati, US | **-** | **P85567** | **P85568** | **P85569** | P85570 | **P85571** |
| *Cryptocercus kyebangensis* | Y.C. Park, ROK | **-** | **P85572** | **P85573** | **P85574** | P85575 | **P85576** |
| *Cyrtotria poduriformis* | Cape Town, ZA | **P84656** | **P85577** | **P85578** | **P85579** |  |  |
| *Derocalymma cruralis* | Ai-Ais, NAM | **P85580** | **P85581** | **P85582** | **P84672** | **submitted** | **submitted** |
| *Derocalymma versicolor* | Kamieskroon, ZA | **P84659** | **P84660** | **P84663** | **P84671** | **submitted** | **submitted** |
| *Deropeltis erythrocephala* | Cape Town, Garies, ZA | P85583 | P84380 | P84380 | P85584 | P85585 | **P85586** |
| *Deropeltis atra* | Port Nolloth, ZA | P85593 | P85594 | P85595 | P85596 | **P85597** | **P85598** |
| *Deropeltis integerrima* | R. Dusi, GER | P85587 | P85588 | P85589 | P85590 | **P85591** | **P85592** |
| *Diploptera punctata* | R. Dusi, GER | **P85599** | **P85600** | **P85601** | P84665 | **P85602** | **P85603** |
| *Elliptorhina spec.* | Institute of Zoology Jena, GER | **P85604** | **P85605** | **P85606** | **P85607** | **P85851** | **P85852** |
| *Ergaula capucina* | R. Dusi, GER | **P85608** | **P85609** | **P85610** | **P85611** | P85612 | **P85613** |
| *Eublaberus distanti* | R. Dusi, GER | **P85614** | **P85615** | **P85616** | **P85617** | **P85618** | **P85619** |
| *Eublaberus prosticus* | R. Dusi, GER | **P85620** | **P85621** | **P85622** | **P85623** | **P85624** | **P85625** |
| *Eublaberus spec.* | Celestun, MEX | **P85626** | **P85627** | **P85628** | **P85629** | **P85630** | **P85631** |
| *Eurycotis floridana* | Institute of Zoology Jena, GER | P85632 | P84375 | P84375 | P85633 | **P85634** | **P85847** |
| *Gromphadorhina portentosa* | Institute of Zoology Jena, GER | **P83925** | **P83930** | **P83935** | **P85641** | P84221 | **P85642** |
| *Gromphadorhina grandidieri* | R. Dusi, GER | **P85635** | **P85636** | **P85637** | **P85638** | **P85639** | **P85640** |
| *Gyna caffrorum* | Cape Vidal, ZA | **P85643** | **P85644** | **P85645** | **P85646** | **P85849** | **P85850** |
| *Gyna lurida* | R. Dusi, GER | **P85647** | **P85648** | **P85649** | **P85650** | **P85651** | **P85652** |
| *Hostilia carinata* | Tsitsikamma, ZA | **P85793** | **P85794** | **P85795** | **P85796** |  |  |
| *Laxta spec.* | Queensland, AUS | **P85653** | **P85654** | **P85655** | **P85656** |  |  |
| *Loboptera decipiens* | H. Bohn, Funjal, P | **-** | **P85657** | **P85658** | **P85659** | **P85846** | **submitted** |
| *Lucihormetica grossei* | R. Dusi, GER | **P85660** | **P85661** | **P85662** | **P85663** | **P85664** | **P85665** |
| *Lucihormetica subcincta* | R. Dusi, GER | **P85666** | **P85667** | **P85668** | **P85669** | **P85670** | **P85671** |
| *Lucihormetica verrucosa* | R. Dusi, GER | **P85672** | **P85673** | **P85674** | **P85675** | **P85676** | **P85677** |
| *Mastotermes darwiniensis* | R. Plarre, GER | **P85678** | **P85679** | **P85680** | **P85681** | P85682 | **P85683** |
| *Neostylopyga rhombifolia* | Institute of Zoology Jena, GER | P85684 | P84428 | P84440 | P85685 | P85848 | **P85686** |
| *Panaesthia spec.* | Queensland, AUS | **P85687** | **P85688** | **P85689** | **P85690** | **P85691** | **P85692** |
| *Panchlora spec*. | Palenque, MEX | **P85693** | **P85694** | **P85695** | **P85696** | **P85697** | **P85698** |
| *Panchlora viridis* | Institute of Zoology Jena, GER | P84658 | P85699 | P85700 | P84670 | **P85853** | **P85854** |
| *Periplaneta americana* | Institute of Zoology Jena, GER | P41837 | P84422 | P81555 | P82617 | P84259 | P36885 |
| *Periplaneta australasiae* | Institute of Zoology Jena, GER | P85701 | P84424 | P84436 | P8570 | **P85703** | **P85704** |
| *Periplaneta brunnea* | Institute of Zoology Jena, GER | P85709 | P84423 | P84435 | P85710 | **P85711** | **P85712** |
| *Periplaneta fuliginosa* | Institute of Zoology Jena, GER | P85713 | P84425 | P84437 | P85714 | **P85715** | **P85716** |
| *Perisphaeria aff. bicolor* | Western Cape, ZA | **P85705** | **P85706** | **P85707** | **P85708** | **submitted** | **submitted** |
| *Perisphaeria ruficornis* | Tsitsikamma, ZA | **P85717** | **P85718** | **P85719** | **P85720** | **P85721** | **P85722** |
| *Perisphaeria scabrella* | Vanrhynsdorp, ZA | **P85723** | **P85724** | **P85725** | **P85726** | **submitted** | **submitted** |
| *Perisphaeria substylifera* | Kareedouw, ZA | **P85727** | **P85728** | **P85729** | **P85730** | **submitted** | **submitted** |
| *Perisphaeria virescens* | Gansbaai, ZA | **P84657** | **P85731** | **P85732** | **P85733** | **submitted** | **submitted** |
| *Pilema dubia* | Sommerset East, ZA | **P85734** | **P85735** | **P85736** | **P85737** |  |  |
| *Polyphaga aegyptiaca* | R. Dusi, GER | **P85738** | **P85739** | **P85740** | **P85741** | P85742 | **P85743** |
| *Princisia vanwaerenbeki* | R. Dusi, GER | **P85744** | **P85745** | **P85746** | **P85747** | **P85748** | **P85749** |
| *Pseudoderopeltis bimaculata* | Kareedouw, ZA | P85750 | P84434 | P84441 | P85751 | **P85752** | **P85753** |
| *Pseudoderopeltis flavescens* | Craddock, ZA | P85754 | P84377 | P84377 | P85755 | **P85756** | **P85757** |
| *Pseudoderopeltis foveolata* | Vanrhynsdorp, ZA | P85758 | P84376 | P84376 | P85759 | **P85760** | **P85761** |
| *Pycnoscelus surinamensis* | Institute of Zoology Jena, GER | **P84654** | **P85762** | **P85763** | **P84667** | **P85764** | **P85765** |
| *Rhyparobia maderae* | Institute of Zoology Jena, GER | P85766 | P85767 | P85768 | P85769 | P85770 | P85771 |
| *Shelfordella lateralis* | R. Dusi, GER | P85772 | P84427 | P84439 | P85773 | **P85855** | **P85855** |
| *Supella dimidiata* | Tsumeb, NAM | **P85774** | **P85775** | **P85776** | **P85777** | **submitted** | **submitted** |
| *Supella longipalpa* | Institute of Zoology Jena, GER | **P85778** | **P85779** | **P85780** | **P85781** | **submitted** | **submitted** |
| *Symploce pallens* | Kareedouw, ZA | **-** | **P85782** | **P85783** | **P85784** | **P85785** | **P85786** |
| *Therea petiveriana* | R. Dusi, GER | **P85787** | **P85788** | **P85789** | **P85790** | P85791 | **P85792** |
| *Drosophila melanogaster* | Gene-Bank | CG 15520 | | | | CG1171 | CG18090 |
| *Locusta migratoria* | Kareedouw, ZA | **P83382** | **submitted** | **submitted** | **submitted** | **submitted** | **submitted** |
